# Two Disaccharide-Bearing Polyethers, K-41B and K-41Bm, Potently Inhibit HIV-1 via Mechanisms Different from That of Their Precursor Polyether, K-41A

**DOI:** 10.3390/cimb46120805

**Published:** 2024-11-25

**Authors:** Jie Liu, Qiuyu Wei, Xin Liu, Jiang Chen, Yujie Zhan, Qinglian Li, Qian Wang, Bingyu Liang, Junjun Jiang, Fengxiang Qin, Zongxiang Yuan, Qiuzhen Qin, Xuehua Li, Yangping Li, Hao Liang, Li Ye, Bo Zhou

**Affiliations:** 1 Guangxi Key Laboratory of AIDS Prevention and Treatment, School of Public Health, Guangxi Medical University, Nanning 530021, China; 2 Joint Laboratory for Emerging Infectious Diseases in China (Guangxi)-ASEAN, Life Sciences Institute, Guangxi Medical University, Nanning 530021, China; 3 Collaborative Innovation Centre of Regenerative Medicine and Medical BioResource Development and Application Co-Constructed by the Province and Ministry, Guangxi Medical University, Nanning 530021, China; 4 CAS Key Laboratory of Tropical Marine Bio-Resources and Ecology, Guangdong Key Laboratory of Marine Materia Medica, RNAM Center for Marine Microbiology, South China Sea Institute of Oceanology, Chinese Academy of Sciences, Guangzhou 510301, China

**Keywords:** HIV-1, antiviral, marine natural products, polyether antibiotics

## Abstract

The screening of novel antiviral agents from marine microorganisms is an important strategy for new drug development. Our previous study found that polyether K-41A and its analog K-41Am, derived from a marine Streptomyces strain, exhibit anti-HIV activity by suppressing the activities of HIV-1 reverse transcriptase (RT) and its integrase (IN). Among the K-41A derivatives, two disaccharide-bearing polyethers—K-41B and K-41Bm—were found to have potent anti-HIV-1_IIIB_ activity in vitro. This study aimed to clarify whether K-41B and K-41Bm have inhibitory effects on different HIV-1 strains or whether these two derivatives have mechanisms of action different from that of their precursor, K-41A. An anti-HIV-1 assay indicated that K-41B and K-41Bm have potent anti-HIV-1_BaL_ activity, with low 50% inhibitory concentrations (IC_50_s) (0.076 and 0.208 μM, respectively) and high selective indexes (SIs) (58.829 and 31.938, respectively) in the peripheral blood mononuclear cell (PBMC)-HIV-1_BaL_ system. The time-of-addition (TOA) assay indicated that K-41B and K-41Bm may exert antiviral effects by activating multiple stages of HIV-1 replication. A cell protection assay indicated that the pretreatment of cells with K-41B or K-41Bm has almost no inhibitory effect on HIV-1 infection. A virus inactivation assay indicated that pretreatment of the virus with K-41B or K-41Bm inhibits HIV-1 infection by 60%. A cell–cell fusion assay showed that K-41B and K-41Bm blocked the cell fusion mediated by viral envelope proteins. The HIV-1 key enzyme experiment also indicated that both compounds have certain inhibitory effects on HIV-1 IN. Furthermore, molecular docking showed that K-41B and K-41Bm interact with several viral and host proteins, including HIV-1 IN, an envelope protein (gp120), a transmembrane protein (gp41), and cell surface receptors (CD4, CCR5, and CXCR4). Overall, in addition to having a similar anti-HIV-1 mechanism of inhibiting HIV-1 IN like the precursor polyether K-41A, the disaccharide-bearing polyether derivatives K-41B and K-41Bm may also inhibit viral entry. This suggests that they display anti-HIV-1 mechanisms that are different from those of their precursor polyethers.

## 1. Introduction

HIV infection remains a major challenge to human health. By 2023, there were 39.9 million people living with HIV-1, with 1.3 million new infections and 630,000 AIDS-related deaths worldwide in 2023 [1]. Although highly active antiretroviral therapy (HAART) has greatly reduced AIDS-related mortality, current therapies have failed to cure HIV infection, with the problems of toxic side effects, drug resistance, and high price (lifetime medication). Therefore, the development of novel antiviral strategies and new agents has been a focus in the field of HIV research.

Marine biogenic natural products are a hot field for drug discovery due to their high diversity, unique structures, and generally lower toxicity than chemically synthesized compounds. According to Marinlit, a marine natural product database, more than 1200 new natural products from marine biological sources are discovered each year. Meanwhile, many secondary metabolites have been found to have certain biological activities, such as anti-inflammatory, antitumor, antibacterial, antiviral, antimalarial, and antioxidant [2,3,4]. Therefore, the search for effective and cheap antiviral drugs from the natural products of marine organisms has become a way to find new anti-HIV drugs. In fact, a number of studies have found some promising marine-derived anti-HIV-1 drugs. For example, truncateols O and truncateols P, the components derived from sponge companion fungus *Truncatella angustata*, have antiviral effects in HIV-1 replication [5]. Baculiferin F-G of the sponge *I. baculifera* from the South China Sea and Da-1 and ACDA-1 in dichloromethane/methanol extracts from Brazilian seaweed inhibit HIV-1 reverse transcriptase (RT), thereby inhibiting viral replication [6]. Phlorotannin derivative 4‴-dieckol from Korean brown alga ecklonia cava not only inhibits the activity of HIV-1 RT but also blocks HIV-1 entry [7]. These studies suggest that marine natural products with similar structures may have similar biological activities, but their antiviral mechanisms may be different. In addition, it is interesting to note that natural marine products may have a multi-target inhibitory effect on HIV-1, which is similar to the drug combination used in HAART therapy to inhibit viral replication more effectively.

Previously, we found that polyether antibiotic K-41A and its analog K-41Am isolated from a marine-derived *Streptomyces* sp. SCSIO 01680 exhibited anti-HIV activity by suppressing the activities of HIV-1 RT and its integrase (IN) [8]. Because the K-41A structure has more O-methylation modifications than other polyether antibiotics, and there is increasing evidence that methylation modifications play a role in HIV infection and disease progression [9,10,11], we speculated that the methylation of K-41A may be closely related to its physicochemical properties and biological functions, including anti-HIV-1 activities. Therefore, K-41A was subjected to a combinatorial biosynthetic study, and nine demethylated and/or deglycosylated derivatives were produced by K-41A methyltransferase gene mutants [12]. Remarkably, in vitro bioassays revealed that two disaccharide-bearing polyethers, K-41B and K-41Bm, display enhanced anti-HIV-1_IIIB_ (a T-tropic viral strain) activity, even superior to the precursor compound K-41A [8,12]. Thus, K-41B and K-41Bm may represent excellent anti-HIV clues for further investigations.

Previous studies have revealed that the anti-HIV-1 mechanisms of different polyether antibiotics were different, and a compound may inhibit HIV replication via several mechanisms [13,14,15]. K-41A, the lead compound of K-41B and K-41Bm, also inhibited both HIV-1 RT and IN activities in our previous study [8]. Simultaneously, numerous studies have indicated that the glycosylation structure is crucial for inhibiting HIV replication against HIV-1 RT, HIV-1 IN, CCR5, etc. [16,17,18,19]. Yarmolinsky et al. found that the 3-O-glycosylation of quercetin and kaempferol significantly improved the inhibitory activity against HSV-1/2 [20]. Research by Pratima Rawat et al. indicated that trehalose, a nonreducing disaccharide, can inhibit macrophages and T cells infected with HIV in a dose-dependent manner, and trehalose decreases viral entry through the downregulation of CCR5 in T cells [16]. The work conducted by Xiaoyang Guan et al. also highlighted the potential importance of glycosylation as an alternative strategy for developing CCR5 inhibitors to inhibit HIV-1 infection [17]. Additionally, numerous studies have indicated that glycosylation plays a significant role in HIV-neutralizing antibodies [21,22]. It is hypothesized that glycosylation modification may lead to K-41B and K-41Bm, exhibiting different anti-HIV activities and mechanisms compared to their precursor compound K-41A.

In this study, we clarified the anti-HIV-1 activities of these two compounds via an ex vivo experiment using an MΦ-tropic viral strain to establish PBMC-HIV-1_BaL_ systems and further explored their potential anti-HIV-1 mechanisms to clarify whether K-41B and K-41Bm have inhibitory effects on different HIV-1 strains or whether the two disaccharide-bearing polyether derivatives have mechanisms different from that to their precursor.

## 2. Materials and Methods

### 2.1. Compounds

The compounds K-41B and K-41Bm, which were derivatives of the polyether K-41A isolated from the marine-derived *Streptomyces* sp. SCSIO 01680 [8,12], were provided by the South China Sea Institute of Oceanology, Chinese Academy of Sciences. Specifically, K-41B and K-41Bm were obtained from the Δpak20 mutant of *Streptomyces* sp. SCSIO 01680 and have been characterized as a known polyether [23] and a C29-methyl ether derivative of K-41B, respectively [12]. The structure information of K-41B and K-41Bm are shown in Table 1. Reference anti-HIV-1 compounds (AZT, NVP, RAL, LPV, T-20) were purchased from Sigma-Aldrich (USA). All compounds were dissolved in dimethyl sulfoxide (DMSO) and diluted to appropriate concentrations with cell culture medium. In our experiments, the highest concentration of DMSO was lower than 0.1%, at which point there was no cytotoxicity in the cells used in this study.

### 2.2. Cells and Viruses

Three cell types (TZM-bl cells, MT-2 cells, and PBMCs) and two HIV-1 strains (X4 tropic HIV-1_IIIB_ and R5 tropic HIV-1_BaL_) were used to establish three HIV-1-cell infection models (TZM-bl-HIV-1_IIIB_, MT-2-HIV-1_IIIB_, and PBMC-HIV-1_BaL_ systems) for experiments in vitro or ex vivo [8]. PBMCs were isolated from healthy donor blood samples by density gradient centrifugation using Ficoll-Hypaque (GE Healthcare, Uppsala, Sweden). The procedures of cell culture and virus infection refer to our previous publication [8].

### 2.3. Cell Viability Assay

Cell viability was assessed using the ATP-based CellTiter-Glo Luminescent Cell Viability Assay (Promega-Beijing, China) as previously described [8,24]. In brief, the cells were seeded in 96-well microtiter plates (2 × 10^5^ cells/well). The cells were treated with serially diluted compounds and incubation for 120 h. The 50% cytotoxic concentration (CC_50_) values were calculated using non-linear regression analysis and dose-response inhibition curves.

### 2.4. Anti-HIV-1 Activity Assay

The antiviral effects of the compounds were evaluated in parallel with a cell viability assay in PBMC-HIV-1_BaL_ system. HIV-1 positive controls were HIV-1-infected cells (no compound treatment), and negative controls were cells that were not infected with HIV-1 (no compound treatment). Azidothymidine (AZT), a known anti-HIV drug, was used as a positive drug control. Each compound was five-fold serially diluted to six concentrations.

In detail, PBMCs isolated from healthy donors were stimulated with phytohemagglutinin (PHA)-P (5 µg/mL) for 72 h, then infected with HIV-1_BaL_ (100 TCID_50_) and treated with compounds. After 120 h, HIV-1 p24 levels in the supernatant were detected using an HIV-1 p24 ELISA kit (Hebei Medical University Biomedical Engineering Centre, Shijiazhuang, China). The 50% inhibitory concentration (IC_50_) values were calculated using non-linear regression analysis and dose-response inhibition curves. The selective index (SI) was calculated based on the ratio of CC_50_ to IC_50_ (SI = CC_50_/IC_50_) to evaluate the potential anti-HIV-1 activities of the compounds.

### 2.5. Anti-Syncytia Assay

Simultaneously, the anti-HIV-1 activity was evaluated via anti-syncytia assay by observing cell morphology in MT-2-HIV-1_IIIB_ system. MT-2 cells (3.2 × 10^4^ cells/well in 96-well microtiter plates) were infected with HIV-1_IIIB_ virus (100 TCID_50_), and serially diluted compounds were added to the cultures. After 72 h, the HIV-1-induced cytopathic effect (CPE) was observed using a microscope. Results are expressed as four levels referring to previous studies [25], including negative (−), weakly positive (+), positive (++), and strongly positive (+++).

### 2.6. Time-of-Addition (TOA) Assay

TOA assay is used to roughly determine the stage of the HIV-1 lifecycle at which a compound inhibits HIV replication, which was performed on a TZM-bl-HIV-1_IIIB_ system. TOA assay was performed according to previous studies [26,27]. Briefly, different anti-HIV-1 drugs (T-20, AZT, RAL, and LPV) with known anti-HIV-1 mechanisms were used as the reference compounds. TZM-bl cells (1 × 10^4^ cells/well) were seeded in 96-well plates and cultured for 24 h, then the cells were infected with HIV-1_IIIB_ (100 TCID_50_). The diluted compounds were added to the culture at the serial time points (0, 2, 4, 6, 8, 10, 12, 18, and 24 h) post-HIV-1 infection. After 48 h, the levels of HIV-1 were measured for luciferase activity using the Promega Bright-Glo Luciferase Assay (Promega-Beijing, China).

### 2.7. Cell Protection Assay

In a cell protection assay, the cells were pretreated with a compound for 1 h before infection to clarify whether the compound had a protective effect on HIV-1 infection of cells. Briefly, TZM-bl cells (1 × 10^4^ cells/well) were cultured in 96-well plates for 24 h. The serially diluted compounds were added to the cultures and incubated at 37 °C for 1 h. Then, the cells were washed with PBS three times to remove the compounds in the cultures and infected with HIV-1_IIIB_ virus (100 TCID_50_). After 48 h, the levels of HIV-1 were measured using a luciferase assay, as described above.

### 2.8. Virus Inactivation Assay

In a virus inactivation assay, HIV-1 viruses were pretreated with a compound for 1 h before infection to determine whether the compound interacts with the virus. Briefly, serially diluted compounds were mixed with HIV-1_IIIB_ (100 TCID_50_) before infection. The mixture was incubated at 37 °C for 1 h, and the compound was removed by ultracentrifugation. Then, the virus was transferred to the TZM-bl cell culture and incubated for 48 h. Triton X-100 is a nonionic surfactant that effectively inactivates lipid-coated viruses and is therefore used as a positive compound control. The levels of HIV-1 were measured using a luciferase assay, as described above.

### 2.9. Cell–Cell Fusion Assay Mediated by HIV-1 Envelope Protein

A cell–cell fusion assay was performed using fluorescently labeled H9-HIV-1_IIIB_ and MT-2 cells as previously described [27]. Briefly, H9-HIV-1_IIIB_ cells in logarithmic growth were collected and adjusted to 1 × 10^6^ cells/mL cell suspension. Calcein-AM was added to H9-HIV-1_IIIB_ cell suspension with a final concentration of 2.5 μM. The cells were incubated at 37 °C for 30 min and washed twice with PBS. The labeled cells were seeded in 96-well plates (1 × 10^4^ cells/well), and the diluted compounds were added to the cultures and incubated for 30 min. Then, MT-2 cells (1 × 10^5^ cells/well) were added to the culture and incubated for 2 h. Cell–cell fusion was observed using an inverted fluorescence microscope. Three control groups were set up. The negative control group was the co-culture of normal H9 and MT-2 cells, the positive control group was the co-cultured cells without compound treatment, and the compound control group was the co-cultured cells treated with T-20.

### 2.10. HIV-1 Key Enzyme Inhibition Assay

HIV-1 key enzyme inhibition assays were carried out using the commercial kits as previously described [8,28]. The inhibition assays on HIV-1 RT, IN, and PR were conducted using Reverse Transcriptase Assay kit (Roche, 11468120910, Mannheim, Germany), HIV-1 Integrase Assay Kit (XpressBio, EZ1700, Frederick, MD, USA), and HIV-1 Protease Inhibitor Screening Kit (Fluorometric, ab211106, Abcam, Shanghai, China), respectively, according to the manufacturer’s instructions. For assays on RT and IN, the detailed procedures can refer to our previous publication [8]. For assay on PR, briefly, the compound, HIV-1 Protease Enzyme Solution, and HIV-1 Protease Substrate Solution were added to a black transparent flat-bottom 96-well plate. A control without compound and the control of the standard drug of Pepstatin A were set simultaneously. The fluorescence intensity values (RFU) at Ex/Em = 330/450 nm were detected at 37 °C in kinetic mode (the samples were measured every 10 min for 3 h) using a microplate reader (BioTek, Synergy H1, Winooski, UK). The slope was calculated from the fitted curve and used to calculate the relative inhibition level.

### 2.11. Molecular Docking

The semi-flexible docking of the compounds and the possible targeted viral proteins or host proteins were performed using Autodock 4.2 software. ChemOffice 2019 was used to map the chemical structures of the compounds and convert them into 3D structures, which were then converted into an Autodock recognizable format using Open Babel. The crystal structures of HIV-1 and the target-related proteins were obtained from the PBD database http://www1.rcsb.org/ (accessed on 24 January 2021), and water molecules and original ligands in the protein crystal complex were removed with PyMOL 2.1.0 software. The structures of the ligand and receptor were optimized using AutoDockTools-1.5.6 and stored in pdbqt format. Subsequently, the docking parameters were set as required, and then the dockings were performed. The conformation with the lowest binding free energy was selected by an Autodock Analyze module, and the binding mode was visualized with PyMOL in 3D. The 2D diagram of ligand–receptor interaction was produced with Discovery Studio 4.5.

## 3. Results

### 3.1. Anti-HIV-1 Activities of K-41B and K-41Bm

**Figure 1:**
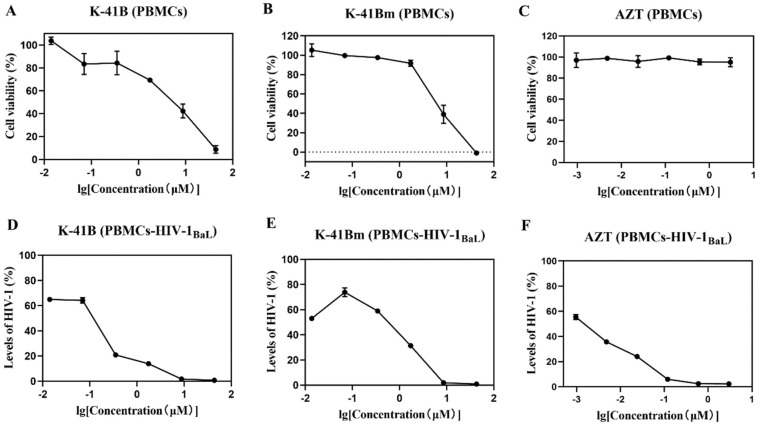
Cytotoxicity and anti-HIV-1 activities of K-41B and K-41Bm in PBMC-HIV-1_BaL_ system. (**A**–**C**) The effects of K-41B (**A**), K-41Bm (**B**), and AZT (**C**) on cell viabilities were measured in parallel mock-infected cells using CellTiter-Glo Luminescent cell viability assay, respectively. (**D**–**F**) The effects of K-41B (**D**), K-41Bm (**E**), and AZT (**F**) on HIV-1 replication, respectively. The levels of HIV-1_BaL_ in the culture supernatants were determined with an HIV-1 p24 ELISA kit. The relative cell viabilities in compound-treated groups were presented as the percentage of control (without compound treatment, which was defined as 100%). The levels of relative HIV-1 replication were presented as the percentage of control (with HIV-1 infection, without compound treatment, which was defined as 100%). AZT treatment was used as a drug control.

The anti-HIV-1 activities of K-41B and K-41Bm were evaluated in PBMC-HIV-1_BaL_ systems (Figure 1 and Table 2). K-41B and K-41Bm exhibited dose-dependent inhibitory effects on HIV-1 replication, and the IC_50_ values were 0.076 and 0.208 μM, respectively. K-41B and K-41Bm showed strong anti-HIV-1 activity, with high SIs of 58.829 and 31.938, respectively.

### 3.2. Anti-Syncytia Activities of K-41B and K-41Bm

**Figure 2:**
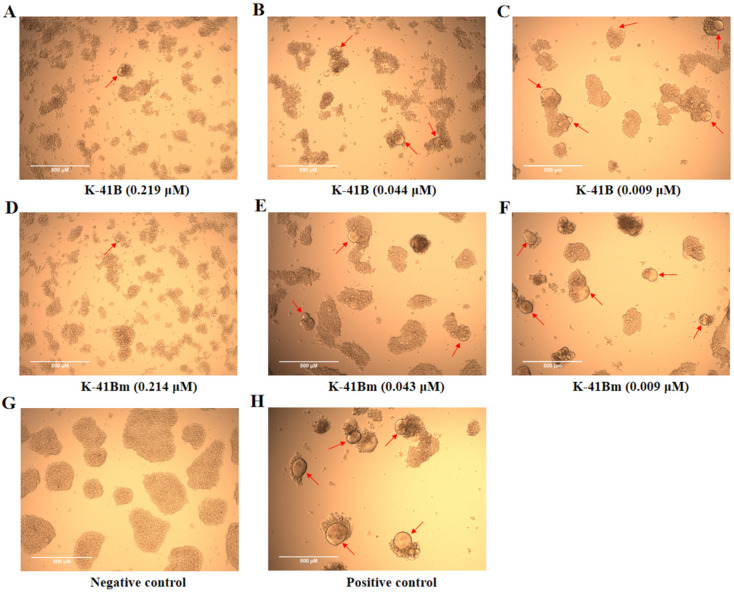
The effects of K-41B and K-41Bm on HIV-1-induced syncytia in MT-2 cells. MT-2 cells were infected with HIV-1_IIIB_ virus and treated with K-41B and K-41Bm. The formation of HIV-1-induced syncytia (indicated by the red arrows) was observed with a microscope at 72 h post-infection. (**A**–**C**) HIV-1-infected cells were treated with different concentrations of K-41B. (**D**–**F**) HIV-1-infected cells were treated with different concentrations of K-41Bm. (**G**) Negative control. MT-2 cells without HIV-1 infection, without compound treatment. (**H**) Positive control. MT-2 cells with HIV-1 infection, without compound treatment.

In terms of cell morphology, as shown in Figure 2, K-41B and K-41Bm reduced the formation of HIV-1-induced syncytia to varying degrees at different concentrations. As shown in the figure, at low concentrations, the characteristics of numerous and large syncytia were similar to those of the positive control group. In contrast, the syncytia became smaller at high concentrations, and their numbers decreased. The semi-quantitative levels of CPE induced by HIV-1 are displayed in Table 3. These results demonstrate that K-41B and K-41Bm could inhibit the formation of HIV-induced syncytia in a dose-dependent manner.

### 3.3. TOA Assay of K-41B and K-41Bm in TZM-bl-HIV-1_IIIB_ System

**Figure 3:**
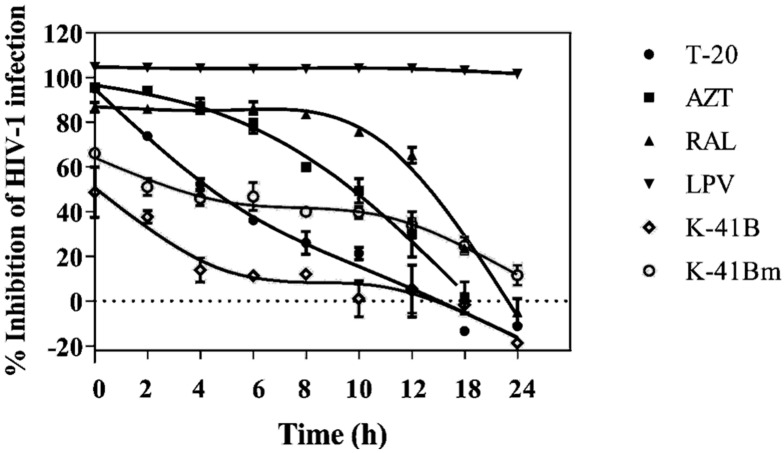
Time-of-addition assay. TZM-bl cells were infected with HIV-1_IIIB_, and the compounds were added to the culture at serial time points (0, 2, 4, 6, 8, 10, 12, 18, and 24 h) post HIV-1 infection, respectively. After 48 h treatment, the levels of HIV-1 were measured by luciferase activity. The percent inhibitory of HIV-1 was calculated using the following formula: [1–(E–N)/(P–N)] × 100%; “E” represents the luciferase value in the experiment group; “N” represents the luciferase value in the negative control group, to which no virus was added; “P” represents the luciferase value in the positive control group, to which no compound was added. The data presented are one representative experiment of repeated experiments. Black circles, T-20 (2.226 μM); squares, AZT (1.496 μM); perpendicular triangle, RAL (4.687 μM); inverted triangle, LPV (79.517 μM); diamond, K-41B (4.653 μM); white circles, K-41Bm (4.600 μM).

A TOA assay was performed using a TZM-bl-HIV-1_IIIB_ system to determine which step(s) of the HIV-1 life cycle are blocked by the compounds (Figure 3). The reference compounds T-20 (virus entry inhibitor), AZT (HIV RT inhibitor), and RAL (HIV IN inhibitor) showed a critical reduction of HIV-1 inhibitory activity at 2, 8, and 12 h, respectively. Additionally, HIV-1 replication was always significantly inhibited by LPV (HIV PR inhibitor) within 24 h post-infection. For K-41B and K-41Bm, their anti-HIV-1 activity was strongest when they were added into cells with HIV-1 synchronously, and then the inhibitory effect gradually decreased, which was similar to T-20. However, the inhibitory effect of both compounds was stable between approximately 6 h and 10 h, and then showed a second decline after 12 h, which was similar to RAL. In general, K-41B and K-41Bm play their antivirus role in the early stages of HIV-1 infection replication.

### 3.4. Cell Protection Assay of K-41B and K-41Bm on TZM-bl Cells

**Figure 4:**
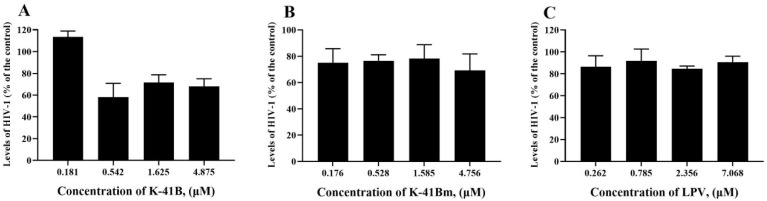
Cell protection assay of K-41B and K-41Bm on TZM-bl cells. TZM-bl cells were pretreated with K-41B (**A**), K-41Bm (**B**), or LPV (**C**) at indicated concentrations for 1 h before HIV-1 infection. Then, the cells were washed with PBS to remove the compounds in the cultures and infected with the HIV-1_IIIB_ virus (100 TCID_50_). At 48 h post-infection, the levels of HIV-1 were measured using a luciferase assay, and the relative HIV-1 levels were presented as a percentage of control (with HIV-1 infection, without compound treatment, which was defined as 100%). Compounds were three-fold serially diluted to four concentrations with LPV as a negative control.

Since the TOA assay showed that K-41B and K-41Bm may inhibit HIV-1’s entry into cells, we carried out a cell protection assay using TZM-bl cells to determine whether K-41B and K-41Bm inhibit HIV-1 infection by interacting with cells. As shown in Figure 4, the pretreatment of cells with K-41B resulted in a 30–40% reduction in HIV infection at a concentration of 0.542–4.875 μM in TZM-bl cells (Figure 4A). However, the pretreatment of cells with K-41Bm had little effect on HIV-1 replication, similar to the control group with LPV (HIV PR inhibitor) pretreatment (Figure 4B,C). Therefore, the pretreatment of cells with K-41B or K-41Bm before exposure to HIV-1 had almost no effect on protecting the cells from HIV-1 infection.

### 3.5. K-41B and K-41Bm Virus Inactivation Assay on HIV-1

**Figure 5:**
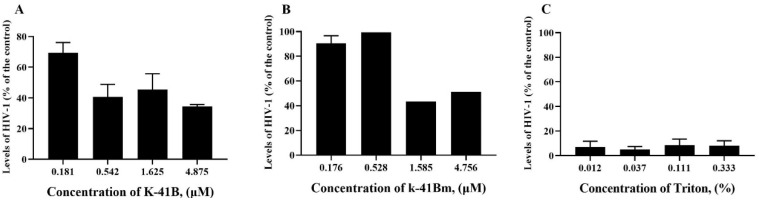
Virus inactivation assay of K-41B and K-41Bm on HIV-1_IIIB_. K-41B (**A**), K-41Bm (**B**), or Triton X-100 (**C**) were premixed with HIV-1_IIIB_ virus (100 TCID_50_) for 1 h. Then, the mixtures were washed with PBS and transferred into the TZM-bl cells culture. At 48 h post-transferring, the levels of HIV-1 were measured using a luciferase assay, and the relative HIV-1 levels were presented as a percentage of control (with HIV-1 infection, without compound treatment, which was defined as 100%). Compounds were three-fold serially diluted to four concentrations with Triton X-100 as a positive drug control.

The inhibition of HIV-1 entry may also be due to the interaction of the compound with the virus. A virus inactivation assay was performed to further analyze whether the anti-HIV-1 activity of the compounds is related to their direct interaction with HIV-1. As shown in Figure 5, the pretreatment of HIV-1 viruses with K-41B or K-41Bm showed a dose-dependent anti-HIV-1 effect in TZM-bl cells, and the highest inhibitory effect was approximately 60% (Figure 5A,B). HIV-1 levels in the Triton X-100-pretreated positive control group remained below 10% at all concentrations, possibly due to the Triton X-100-inactivated viruses sticking to cells (Figure 5C). The results indicate that K-41B and K-41Bm may exert antiviral effects by directly interacting with HIV-1.

### 3.6. Cell–Cell Fusion Inhibition Assay of K-41B and K-41Bm on H9-HIV-1_IIIB_ and MT-2 Cells

**Figure 6:**
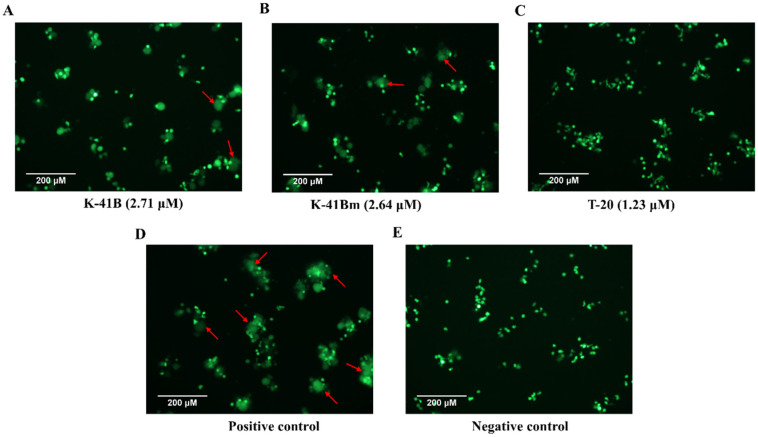
The cell–cell fusion inhibition assay of K-41B and K-41Bm on H9-HIV-1_IIIB_ cells and MT-2 cells. H9-HIV-1_IIIB_ cells labeled with Calcein-AM dye were treated with K-41B (**A**), K-41Bm (**B**), or T-20 (**C**) and MT-2 cells. And after 2 h, cell–cell fusion (indicated by the red arrows) was observed using an inverted fluorescence microscope. Three control groups were set, including the reference compound control with T-20 (**C**), the positive control without compound (**D**), and the negative control of normal H9 cells co-cultured with MT-2 cells (**E**).

H9 cells expressed viral envelope proteins post-HIV-1 infection, and the morphology was no different from that of normal H9 cells. However, the fluorescently labeled H9-HIV-1_IIIB_ cells became larger, and the fluorescence intensity became weaker when they were co-cultured with uninfected MT-2 cells for H9-HIV-1_IIIB_ cells fused with MT-2 cells, and the fluorescent dye spread in the fused cells. Therefore, a cell–cell fusion inhibition assay could be used to visually distinguish whether compounds affect HIV-1’s entry into cells. As shown in Figure 6, no cell–cell fusion was found in the wells treated with T-20 or in the negative control wells (Figure 6C,E). However, the fusion of H9-HIV-1_IIIB_ and MT-2 cells showed a significant reduction when treated with K-41B or K-41Bm compared to the positive control group, which showed many confluent cells with weak fluorescence (Figure 6A,B,D). The results indicate that K-41B and K-41Bm can effectively inhibit HIV-1 Env-mediated cell–cell fusion.

### 3.7. Effects of K-41B and K-41Bm on HIV-1 RT, in, and PR Activities

**Figure 7:**
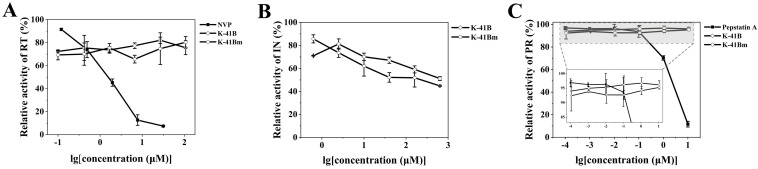
Effects of K-41B and K-41Bm on HIV-1 key enzyme activities. The HIV-1 RT inhibition assay (**A**), HIV-1 IN inhibition assay (**B**), and HIV-1 PR inhibition assay (**C**) were performed using ELISA kits according to the manufacturer, and NVP, sodium azide, and Pepstatin A were used as the positive control, respectively. The relative enzyme activity levels are presented as a percentage of control (without compound treatment, which was defined as 100%). Since the concentration unit (%) of sodium azide differed from that of K-41B and K-41Bm (μM), causing incompatibility, Figure B did not display the result of sodium azide.

To further illuminate the possible mechanism involved in the anti-HIV-1 activity of K-41B and K-41Bm, commercial ELISA kits were used to detect the inhibitory effects of K-41B and K-41Bm on the activities of three key HIV-1 enzymes (RT, IN and PR). As shown in Figure 7, K-41B and K-41Bm inhibited the activity of HIV-1 IN in a dose-dependent manner, and the highest suppression was approximately 50% and 55%, respectively (Figure 7B). The inhibitory activity of K-41B or K-41Bm on HIV-1 RT was maintained at 20~30% (Figure 7A), but neither affected HIV-1 PR (Figure 7C).

### 3.8. Molecular Docking of HIV-1-Related Proteins and Cell Surface Receptors with K-41B and K-41Bm

**Figure 8:**
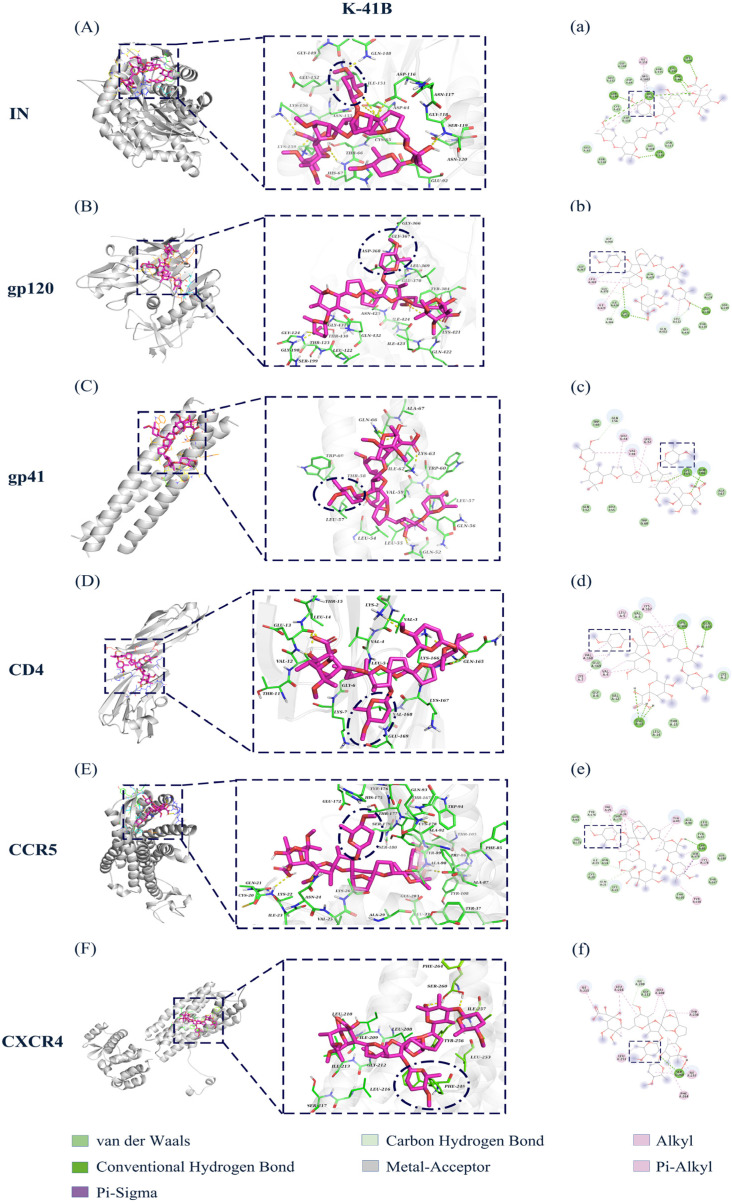
The protein–ligand interactions of K-41B with HIV-1 key proteins. (**A**–**F**) The 3D images of protein–ligand interactions of K-41B (purple) with HIV-1 IN (**A**), gp120 (**B**), gp41 (**C**), CD4 (**D**), CCR5 (**E**), and CXCR4 (**F**). (**a**–**f**) are the corresponding 2D images. The dark blue oval in the 3D image and the dark blue rectangle in the 2D image denote the glycosylated modified structure.

**Figure 9:**
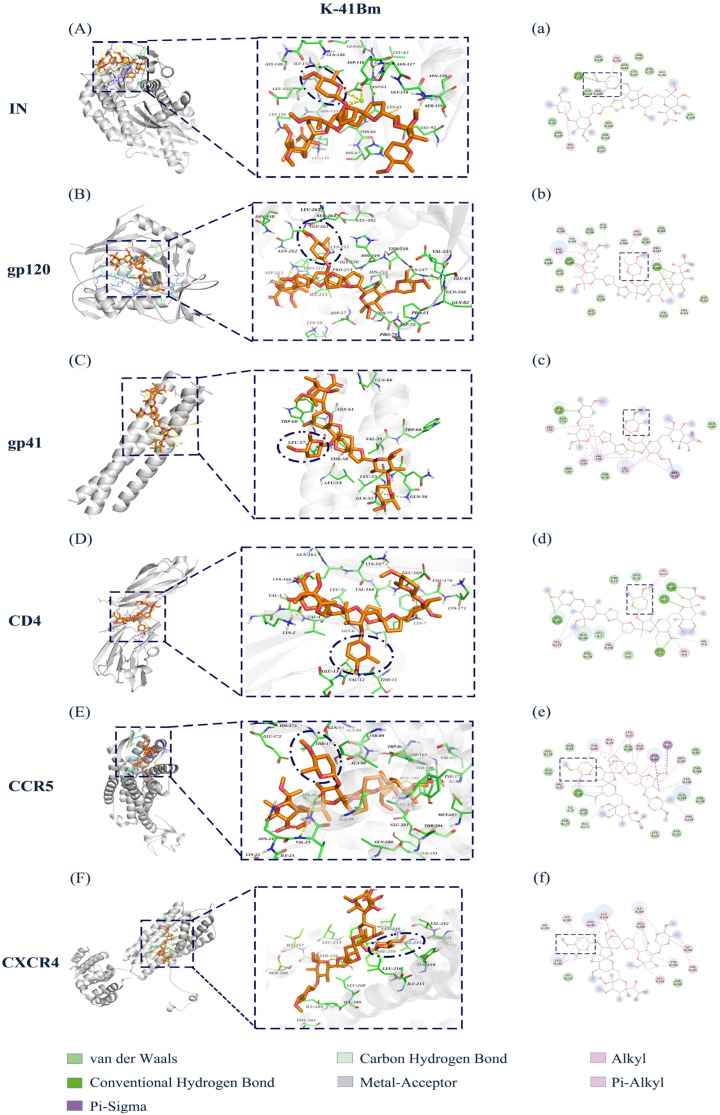
The protein–ligand interactions of K-41Bm with HIV-1 key proteins. A-F show the 3D images of protein–ligand interactions of K-41Bm (orange) with HIV-1 IN (**A**), gp120 (**B**), gp41 (**C**), CD4 (**D**), CCR5 (**E**), and CXCR4 (**F**). (**a**–**f**) are the corresponding 2D images. The dark blue oval in the 3D image and the dark blue rectangle in the 2D image denote the glycosylated modified structure.

Molecular docking is extensively applied in structure–activity relationship (SAR) studies [29,30,31]. To further study the interaction between the compounds and the targets, we analyzed the binding modes of HIV-1-related proteins (HIV-1 IN, gp120, and gp41) and cell surface receptors (CD4, CCR5, and CXCR4) with K-41B (Figure 8) or K-41Bm (Figure 9) using molecular docking. The results showed that both K-41B and K-41Bm interacted with all of the above molecules. The free binding energy between their docking and each target protein was similar, ranging from −3.69 to −7.06 kcal/mol (Table 4). Figure 8 and Figure 9 show 3D and 2D representations of the lowest energy pose of K-41B or K-41Bm docked into the molecules’ binding pockets. The 3D images show that K-41B and K-41Bm could be attached to the active pockets of each target protein, and the action sites of these two compounds are similar. By visualizing the receptor–ligand complexes, it was observed that each compound interacted with specific residues of the protein through various types of bonds, including van der Waals, conventional hydrogen bonds, carbon–hydrogen bonds, alkyl hydrophobic bonds, and mixed Pi/alkyl hydrophobic bonds. Similarly, the binding effects between two glycol groups (C15- and C27-disaccharide structures of K-41B and K-41Bm) and protein residues were also mainly mediated by van der Waals, hydrophobic, and hydrogen bonds. Notably, the C15-glycosylation structure, along with the distinct glycosylation modification patterns of K-41B and K-41Bm compared to A, nearly interacted with each target protein.

## 4. Discussion

The anti-HIV activities displayed by small molecule compounds derived from marine microorganisms attract much attention [32,33,34]. Inspired by the superior anti-HIV-1_IIIB_ (a T-tropic viral strain) effect of K-41B and K-41Bm that we found previously [12], we further researched their anti-HIV-1 activity using HIV-1_BaL_ (an MΦ-tropic viral strain) and explored the potential mechanism. The results showed that K-41B and K-41Bm could inhibit the replication of the HIV-1_BaL_ strain and are very attractive due to their low IC_50_ (0.076 μM and 0.208 μM, respectively) and high SI values (58.829 and 31.938, respectively). In terms of morphology, K-41B and K-41Bm reduced the syncytium formation of MT-2 cells caused by HIV-1_IIIB_. This indicates that there was no significant coreceptor tropism for the anti-HIV-1 activity of K-41B and K-41Bm; in other words, they may have broad-spectrum anti-HIV-1 activity and may be a multi-target HIV-1 inhibitor. Our previous research found that K-41B exhibits stronger anti-HIV-1 activity against HIV-1_IIIB_ than K-41A. Although the anti-HIV activity of K-41B was weakened against HIV-1_BaL_, it achieved high SIs in three different cell infection models, indicating a broader spectrum of anti-HIV-1 activity. The only difference between K-41B and K-41A was the addition of a glycosyl group, which suggests that glycosylation modification significantly influenced the anti-HIV-1 activity. Furthermore, K-41B demonstrated stronger anti-HIV-1 activity than K-41Bm in all three cell-infection models. The only difference between the two was the presence of a hydrogen atom versus a methyl group at the same site, indicating that this site is closely related to their anti-HIV activity; the importance of this site was also confirmed in the results of K-41A and K-41Am. In summary, minor structural changes have a substantial impact on the anti-HIV-1 activity. This study on the anti-HIV-1 activity of K-41B and K-41Bm provides further insights for developing broader-spectrum and more potent structural analogs.

Previous studies have shown that HIV entry mostly occurs within 4 h after exposure and that reverse transcription begins immediately after entry. The integration process is usually completed within 10 h of infection, and a complete intracellular viral replication cycle takes approximately 24 h [35,36,37]. We first conducted a time-of-addition experiment to preliminarily clarify the effect stages of K-41B and K-41Bm in the HIV-1 life cycle. On the whole, the antiviral activity trends of K-41B and K-41Bm were similar to those of the fusion inhibitor T-20 and the integrase inhibitor RAL. Notably, their antiviral activities remained almost unchanged when these two compounds were added within 6 to 10 h of infection. It is speculated that the compound may be involved in the integration process of HIV-1 RNA, so even though the virus cannot be completely eliminated, the compound continues to have an antiviral effect after its blocking effect on other pre-integration processes has diminished. Therefore, it is preliminarily concluded that K-41B and K-41Bm may exert antiviral effects by acting on multiple targets in the early stages of HIV-1 replication.

The early stage of HIV-1 replication mainly includes virus entry, reverse transcription, and integration [38]. First, we conducted cell protection and virus inactivation assays to determine whether K-41B and K-41Bm affect cells or viruses during virus entry. The results revealed that K-41B and K-41Bm had a relatively stronger inhibition effect in the virus inactivation assay, while they showed little anti-HIV-1 activity in the cell protection assay. This suggests that these two compounds primarily directly affected HIV-1 and reduced the infectivity of the virus. The results of the cell–cell fusion assay also showed that the fusion of H9-HIV-1_IIIB_ and MT-2 cells resulted in a significant reduction when treated with K-41B or K-41Bm, indicating that K-41B and K-41Bm could block the cell fusion process mediated by viral envelope proteins. Given the results of the cell–cell fusion assay, we hypothesized that K-41B and K-41Bm might have a protective effect on cells by binding with the CD4 receptor and CCR5/CXCR4 coreceptor to change the conformation of the cell surface to block the virus entering the cells. In addition, previous studies have found that polyether antibiotics may interfere with the absorption of viruses into cells [39], which supports the effect of K-41B and K-41Bm on virus entry. We also conducted the HIV-1 key enzymes experiment and found that both compounds had certain inhibitory effects on HIV-1 IN. The above results indicate that K-41B and K-41Bm may be multi-target HIV-1 inhibitors, which is in accordance with the current trend of anti-HIV drug development.

In recent years, the emergence of computer-aided drug design technology has provided a more convenient, rapid, and efficient way for innovative drug development. As the core technology of virtual screening, molecular docking has been widely used to predict the binding conformation and ability between ligands and receptors [40,41]. The results of molecular docking showed that K-41B and K-41Bm interacted with several protein molecules related to viral replication, including HIV-1 IN, an envelope protein (gp120), transmembrane protein (gp41), and cell surface receptors (CD4, CCR5, and CXCR4). The free binding energy for both of them docking to each target protein was similar, and the binding effect was mainly mediated by van der Waals, hydrophobic, and hydrogen bonds. It was again demonstrated that K-41B and K-41Bm may exert their anti-HIV-1 effects by acting on multi-targets.

In general, in addition to having anti-HIV-1 mechanisms that inhibit HIV-1 IN similar to K-41A and K-41Am, K-41B and K-41Bm may also act as inhibitors during viral entry and exhibit anti-HIV-1 mechanisms different from those of their precursor polyethers. The different anti-HIV-1 mechanisms might be due to the disaccharide structure of K-41B and K-41Bm. The results of the molecular docking showed that the disaccharide structure of K-41B and K-41Bm interacted with viral proteins (gp120 and gp41) and cell surface receptors (CD4, CCR5, and CXCR4), mainly mediating by van der Waals, hydrophobic, and hydrogen bonds. Notably, the additional C15-glycosylation structure in K-41B and K-41Bm played a crucial role in the docking of the two compounds with protein residues, nearly interacting with each protein. In fact, numerous studies have indicated that the glycosylation structure is crucial for developing HIV inhibitors against HIV-1 RT, HIV-1 IN, CCR5, etc. [16,19]. In this study, upon binding to IN, the most prominent interaction of K-41B and K-41Bm was the formation of hydrogen bonds with oxygen in the ortho position of the *O*-glycosylation at C15 and Gln148. In the docking of gp120 with the two compounds, the C15- and C27-disaccharide structures formed hydrogen bonds and alkyl-type bonds with several residues. When docking to CCR5, the C15-glycosylation of K-41B formed a carbon–hydrogen bond with Tyr176, and K-41Bm formed an alkyl–tyle bond with Tyr89. Meanwhile, given the results of the TOA assay, the cell–cell fusion assay, and the HIV-1 key enzymes experiment, we believe that in addition to having a similar anti-HIV-1 mechanism of inhibiting HIV-1 IN like the precursor polyether K-41, the disaccharide-bearing polyether derivatives K-41B and K-41Bm may also inhibit viral entry.

In conclusion, our study found that two disaccharide-bearing polyethers, K-41B and K-41Bm, derived from K-41A, show superior anti-HIV-1 activity and display different anti-HIV-1 mechanisms to their precursor polyether, which might be due to their disaccharide structure as it differs from that of their precursor, K-41A. This study highlights the potential importance of glycosylation in developing HIV inhibitors and provides a scientific basis for the structural modification and optimization of novel anti-HIV lead compounds.

## Figures and Tables

**Table 1:** Chemical structures of K-41B and K-41Bm [12,23].

Compound	Chemical Formula	Chemical Structure
K-41B	C_54_H_92_O_20_	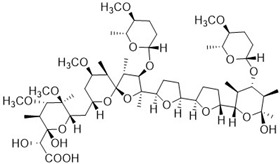
K-41Bm	C_55_H_94_O_20_	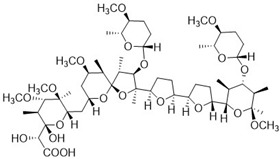

**Table 2:** Cytotoxicity and anti-HIV-1 activities of K-41B and K-41Bm in PBMC-HIV-1_BaL_ systems.

Compound	CC_50_^a^ (μM)	IC_50_^b^ (μM)	SI ^c^
K-41B	4.471	0.076	58.829
K-41Bm	6.643	0.208	31.938
AZT	>2.994	0.002	>1497

^a^ CC_50_: 50% cytotoxic concentration; ^b^ IC_50_: 50% inhibitory concentration. ^c^ SI = CC_50_/IC_50_.

**Table 3:** Inhibition of K-41B and K-41Bm on HIV-1-induced syncytia.

Compound	Concentration (μM)	CPE *
K-41B	0.219	+
	0.044	++
	0.009	+++
K-41Bm	0.214	+
	0.043	++
	0.009	+++
Negative control	/	−
Positive control	/	+++

* Results expressed as four levels, including negative (−), weakly positive (+), positive (++), and strongly positive (+++).

**Table 4:** Summary of binding energy of K-41B and K-41Bm docking to target proteins.

Protein Name	PDB ID	Binding Energy (kcal/mol)	
		K-41B	K-41Bm
HIV-1 IN	1qs4	−3.84	−4.36
HIV-1 gp120	6utd	−3.69	−4.32
HIV-1 gp41	6j5e	−5.05	−4.96
CD4	3o2d	−5.77	−7.06
CCR5	4mbs	−5.64	−6.31
CXCR4	3odu	−4.50	−5.87

## Data Availability

The data presented in this study are available upon reasonable request from the corresponding author.
